# Conversion of *Escherichia coli* to Generate All Biomass Carbon from CO_2_

**DOI:** 10.1016/j.cell.2019.11.009

**Published:** 2019-11-27

**Authors:** Shmuel Gleizer, Roee Ben-Nissan, Yinon M. Bar-On, Niv Antonovsky, Elad Noor, Yehudit Zohar, Ghil Jona, Eyal Krieger, Melina Shamshoum, Arren Bar-Even, Ron Milo

**Affiliations:** 1 Department of Plant and Environmental Sciences, Weizmann Institute of Science, Rehovot 7610001, Israel; 2 Department of Life Sciences Core Facilities, Weizmann Institute of Science, Rehovot 7610001, Israel

**Keywords:** synthetic autotrophy, carbon fixation, Rubisco, adaptive laboratory evolution, synthetic biology, sustainability, metabolic rewiring, *Escherichia coli*

## Abstract

The living world is largely divided into autotrophs that convert CO_2_ into biomass and heterotrophs that consume organic compounds. In spite of widespread interest in renewable energy storage and more sustainable food production, the engineering of industrially relevant heterotrophic model organisms to use CO_2_ as their sole carbon source has so far remained an outstanding challenge. Here, we report the achievement of this transformation on laboratory timescales. We constructed and evolved *Escherichia coli* to produce all its biomass carbon from CO_2_. Reducing power and energy, but not carbon, are supplied via the one-carbon molecule formate, which can be produced electrochemically. Rubisco and phosphoribulokinase were co-expressed with formate dehydrogenase to enable CO_2_ fixation and reduction via the Calvin-Benson-Bassham cycle. Autotrophic growth was achieved following several months of continuous laboratory evolution in a chemostat under intensifying organic carbon limitation and confirmed via isotopic labeling.

## Highlights

• Conversion of obligate heterotroph to full autotrophy over laboratory timescales
• Non-native Calvin cycle operation generates biomass carbon from CO_2_ in *E. coli*
• Formate is oxidized by heterologous formate dehydrogenase to provide reducing power
• Chemostat-based directed evolution led to complete trophic mode change in ≈200 days

## Graphical Abstract

**Figure:**
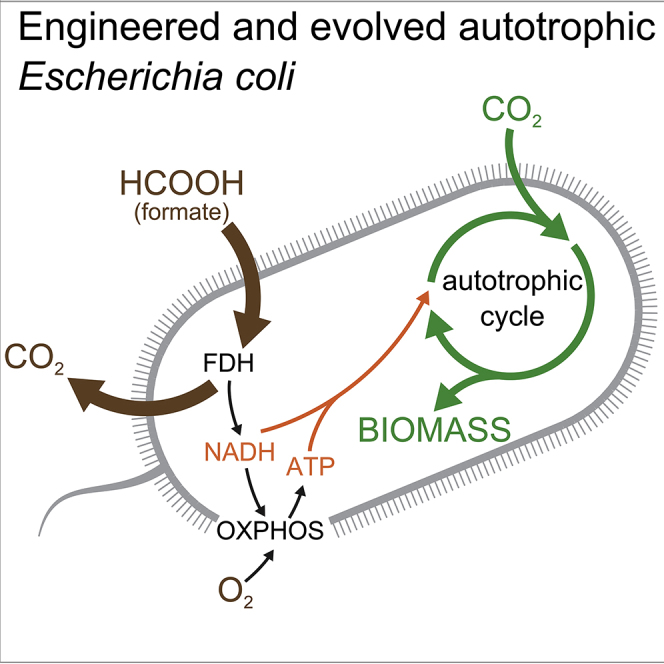

## Teaser

Metabolic rewiring and directed evolution led to the emergence of *E. coli* clones that use CO_2_ as their sole carbon source, while formate is oxidized to provide all the reducing power and energy demands.

## Introduction

Autotrophic organisms, which generate biomass by fixing inorganic carbon into organic compounds, are the main gateway between the inorganic and living worlds. They dominate the biomass on Earth (Crowther et al., 2015, Bar-On et al., 2018), supplying all of our food and most of our fuel. A better understanding of the principles of autotrophic growth (Smith and Stitt, 2007) and methods to enhance it (Ort et al., 2015, Kromdijk et al., 2016, Schwander et al., 2016, South et al., 2019) are thus critical on the path to sustainability. By constructing synthetic autotrophic organisms, we could learn what the main constraints are on natural autotrophs and how to improve their central metabolic pathways. Thus, a grand challenge in synthetic biology is to engineer autotrophy within a model heterotrophic organism.

We can break this formidable task into three essential components. To enable a complete transition to autotrophy, the host must (1) operate CO_2_ fixation machinery in a pathway where the carbon input is comprised solely of CO_2_, while the outputs are organic molecules that enter central carbon metabolism and supply all 12 essential biomass precursors of the cell (Nielsen and Keasling, 2016); (2) express enzymatic machinery to obtain reducing power, either by harvesting non-chemical energy (light, electricity, etc.) or by oxidizing a reduced chemical compound that does not serve as a carbon source; and (3) regulate and coordinate the energy-harvesting and CO_2_-fixation pathways so that they together support steady-state growth with CO_2_ as the sole source of carbon (Barenholz et al., 2017). Previous attempts (Mattozzi et al., 2013, Antonovsky et al., 2016, Schada von Borzyskowski et al., 2018) to establish autocatalytic CO_2_ fixation cycles in model heterotrophs required the addition of multi-carbon organic compounds, which served, at least partially, as a carbon source, in order to achieve stable growth. Specifically, the metabolic design in our previous work (Antonovsky et al., 2016, Herz et al., 2017) was such that CO_2_ was the source of only a third of the cellular biomass carbon, with the rest supplied by an organic acid that served also as the reducing power and energy source. Therefore, the engineering of a heterotrophic organism to supply all its biomass components from inorganic carbon is still a standing challenge.

Here, we report the establishment of synthetic autotrophy in *E. coli.* Our engineered *E. coli* strain uses the Calvin-Benson-Bassham cycle (CBB, also referred to as Calvin cycle for short) for carbon fixation and harvests energy and reducing power from the one-carbon molecule formate (HCOO^−^), which can be produced electrochemically. The stepwise bioengineering process required coexpression of Calvin cycle enzymes and an energy harvesting enzyme, rational rewiring of the endogenous metabolic network, and adaptive laboratory evolution (Sonderegger and Sauer, 2003, Blount et al., 2012, Antonovsky et al., 2016) to achieve the desired trophic mode transformation. The establishment of synthetic autotrophy demonstrates the incredible plasticity of central metabolism and could provide a framework for future carbon-neutral bioproduction.

## Results

### Metabolic Rewiring and Lab Evolution for Conversion to Autotrophy

**Figure 1:**
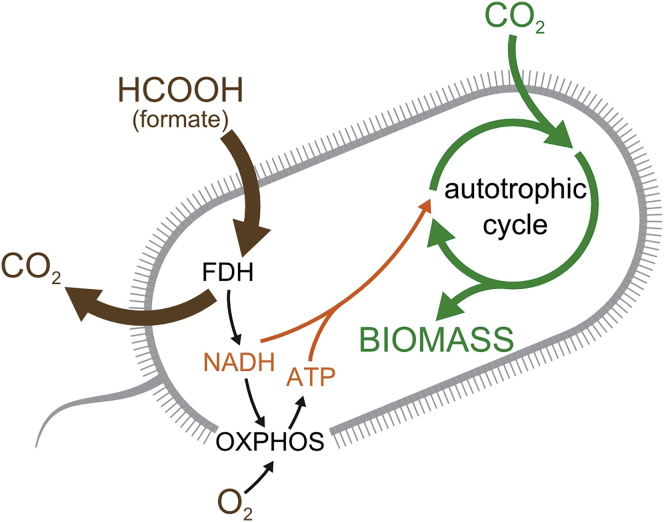
Schematic Representation of the Engineered Synthetic Chemo-autotrophic *E. coli* CO_2_ (green) is the only carbon source for all the generated biomass. The fixation of CO_2_ occurs via an autotrophic carbon assimilation cycle. Formate is oxidized by a recombinant formate dehydrogenase (FDH) to produce CO_2_ (brown) and NADH. NADH provides the reducing power to drive carbon fixation and serves as the substrate for ATP generation via oxidative phosphorylation (OXPHOS in black). The formate oxidation arrow is thicker than the CO_2_ fixation arrow, thus indicating a net CO_2_ emission even under autotrophic conditions. See also Figure S1.

**Figure S1:**
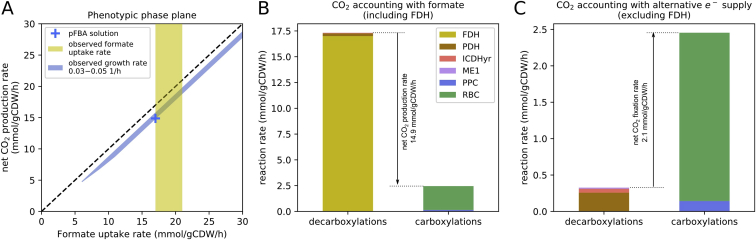
Flux Balance Analysis of the Autotrophic *E. coli*., Related to Figures 1 and 2 (A) Phenotypic phase plane showing the feasible space given the measured growth rate (0.04 ± 0.01 h^-1^) of the evolved strain (blue line). There is strong coupling between the formate uptake and the net CO_2_ production rate since formate can only be metabolised via FDH in our model. In reality, formate can be used for a relatively small flux of C1-related biosynthesis and these reactions are not part of the core model. However, at the measured growth rate, these fluxes are negligible compared to the FDH rate. The yellow shading indicates our measured value for the formate uptake rate (19 ± 2 mmol/gCDW/h). The blue cross indicates the flux balance analysis solution with the minimal total sum of fluxes (also known as pFBA). (B) Stacked bar plot showing the fluxes of all carboxylation and decarboxylation reactions, for the pFBA solution. FDH is by far the most significant decarboxylator, and rubisco is the major carboxylating reaction. (C) Same as B, except that we assume an alternative source for electrons which is CO_2_ neutral (note that the scale of the y axis is different). For example, if formate is produced electrochemically, its contribution to the net CO_2_ would cancel out. Legend abbreviations are as follows: FDH, formate dehydrogenase; PDH, pyruvate dehydrogenase; ICDHyr, isocitrate dehydrogenase; ME1, NAD^+^-dependent malate dehydrogenase; PPC, phosphoenolpyruvate carboxylase; RBC, rubisco.

In order to convert *E. coli* to autotrophy in the laboratory, we considered several candidate compounds (Claassens et al., 2018) to serve as electron donors for CO_2_ fixation. We chose formate as our electron source, because this one-carbon organic compound can serve as a source of reducing power (Berríos-Rivera et al., 2002) but does not naturally support the growth of *E. coli* and is not assimilated into biomass. Its reduction potential (E^0^ = −420 mV) is low enough to reduce NAD^+^, the main electron carrier in the cell (E^0^ = −280 mV under physiological conditions in *E. coli*) (Huang et al., 2012). Another advantage is that it can be electrochemically produced from renewable sources (Yishai et al., 2016) and is seen as a promising path for carbon negative biomass formation. To harvest the electrons from formate and direct them into the main cellular reducing power reservoir NADH, we used an NAD^+^-coupled formate dehydrogenase (FDH; EC 1.17.1.9) from the methylotrophic bacterium *Pseudomonas sp. 101* (Egorov et al., 1980). Stoichiometric analysis of the metabolic network in *E. coli* (Orth et al., 2010b) suggests that the addition of FDH, Rubisco, and phospho-ribulo-kinase (Prk) to the metabolic network of *E. coli* is sufficient for *in silico* autotrophic growth (Volpers et al., 2016) in M9 minimal medium with formate and CO_2_ as cosubstrates (Figure 1). Yet, co-expression of the three recombinant enzymes in a naive BW25113 *E. coli* strain did not result in growth in autotrophic conditions. The stoichiometric analysis (Figure S1) does not take into account requirements such as tuning enzyme kinetics, expression level, and regulation. We thus decided to use adaptive laboratory evolution as a metabolic optimization tool (Antonovsky et al., 2016) to achieve autotrophic growth.

**Figure 2:**
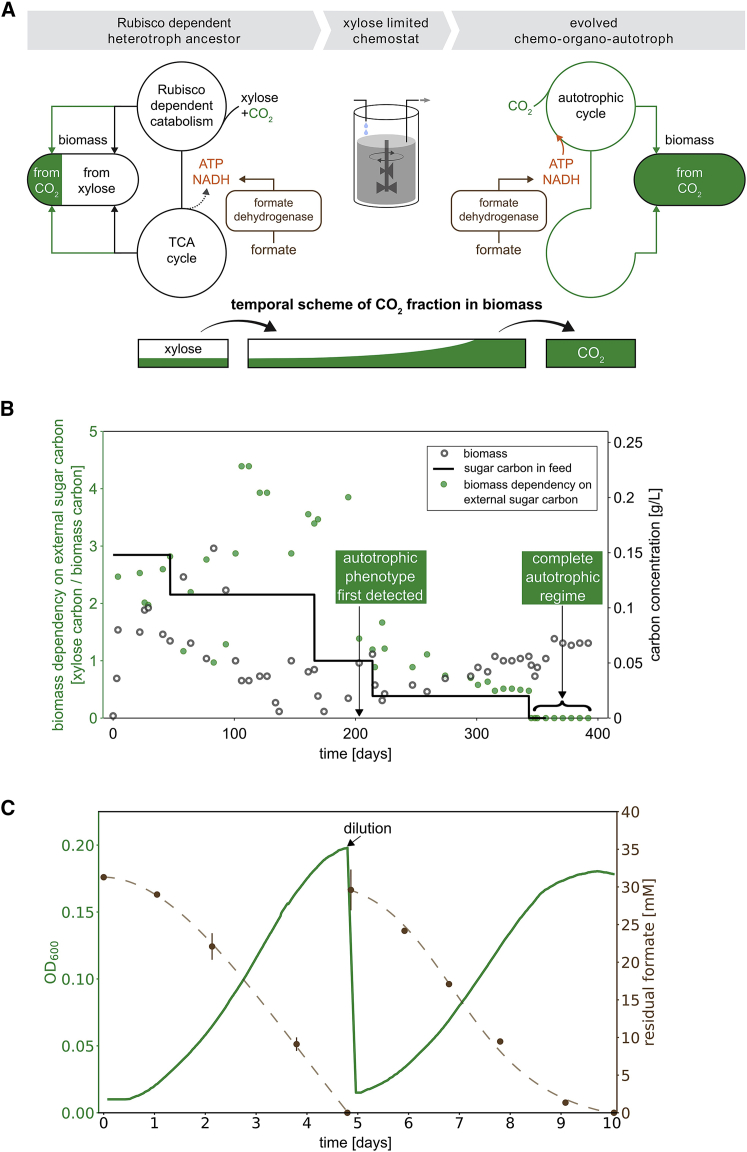
Tailored Evolutionary Strategy from a Rationally Designed Engineered *E. coli* Strain toward an Evolved Chemo-autotroph (A) The parental strain for the evolution (left) harboring knockouts of the pfkAB and zwf genes, and overexpressing Rubisco, Prk, CA, and FDH, assimilates CO_2_ to enable xylose catabolism via the Rubisco-Prk shunt (see also Figure S2) but is unable to grow in autotrophic conditions. Upon xylose starvation in a xylose-limited chemostat with an excess of formate and CO_2_, the cells are under a strong selection pressure to use CO_2_ as the only carbon source, while using formate oxidation by FDH as the energy source. Evolved clones with a fully autotrophic phenotype (right) and a maximal growth rate in the absence of xylose higher than the dilution rate of the chemostat are predicted to have a fitness advantage over xylose-dependent clones and can take over the population. (B) The ancestral strain was inoculated into a xylose-limited chemostat with a dilution rate of 0.02 h^−1^. The concentration of the externally supplied sugar D-xylose in the feed media (black line) was decreased several times throughout the experiment. The biomass dependency on the externally supplied sugar (green dots) decreased starting at day 120, from a value of ≈15 xylose carbons/biomass carbon to zero following day 340 (≈250 chemostat generations). Starting from day 203 (≈150 chemostat generations) of the experiment and onward, we observed that samples taken from the chemostat could grow on minimal media supplemented only with formate and elevated CO_2_. For time points where the culture was not in steady chemostat mode (as described in the STAR Methods), the biomass dependency measure is not shown. (C) Repeated growth of the isolated evolved clone in liquid M9 minimal media with 30 mM sodium formate and sparged with a gas mixture of 10% CO_2_, 90% air. The doubling time of the evolved cells at the given conditions is 18 ± 4 h. Growth was carried out in DASGIP fermenters (150 mL working volume). Residual formate concentrations are represented by brown circles (n = 3, ± SD for values above 8 mM; n = 2, ± SD for values below 8 mM) (see also Figure S3).

**Figure S2:**
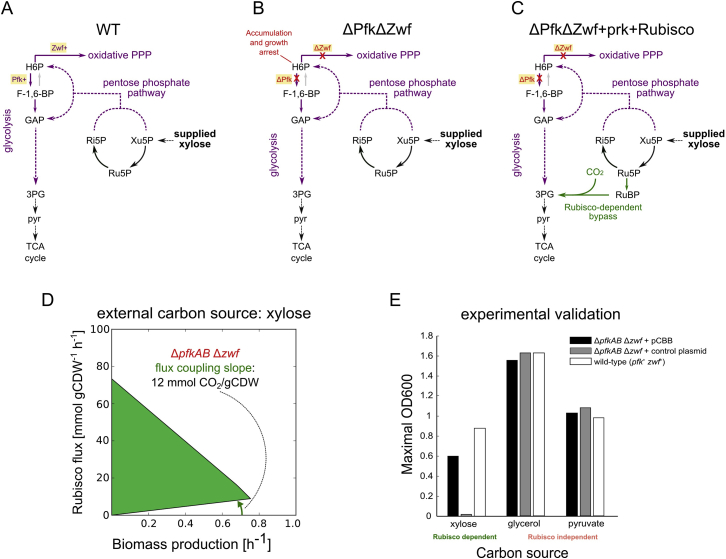
Metabolic Configuration for Mixotrophic Rubisco-Dependent Growth, Related to Figure 2 (A) Metabolic depiction of native route of xylose metabolism in *E. coli* via the pentose phosphate pathway into glycolysis. (B) Knockout of the glycolytic phosphofructokinase (PfK) reaction and glucose-6-phosphate dehydrogenase (Zwf) reaction eliminate the possibility to shunt hexose-phosphates to any oxidative pathways and lead to their accumulation and arrest of growth. (C) Growth of the knockout strain could be rescued upon shunting excess pentose-phosphates via the carbon fixation branch (Prk + Rubisco) into glycolysis. (D) Computational prediction regarding the coupling between carboxylation flux through Rubisco (y axis) and growth (x axis) of the metabolic configuration depicted in (C). (Antonovsky et al., 2016) (E) experimental validation of the Δ*pfkAB*Δ*zwf* metabolic configuration: dependency on the expression of the carbon fixation branch is found only when xylose serves as the single organic carbon (n = 1 for each bar).

The basic rationale behind our approach is as follows: heterologous expression of non-native enzymatic machinery expands the space of possible metabolic reactions for the cell, enabling autotrophic growth. However, this does not guarantee that the needed flux will flow through the newly expanded set of reactions. In fact, as the central metabolism of *E. coli* is adapted to heterotrophic growth, it is likely that flux distribution that supports heterotrophic growth would continue to be utilized. To drive flux toward the desired metabolic pathway, we employed adaptive laboratory evolution. Our approach combines rewiring central metabolism to establish a dependence on the Rubisco carboxylation flux, tailoring the growth medium to inhibit flux through the native heterotrophic pathways, and providing a significant selective advantage to utilizing autotrophic pathways. This, we hypothesized, will lead to the needed tuning of enzyme activity in a way that will divert flux to autotrophic pathways. The way in which our approach was implemented is shown in Figure 2A. First, we knocked out three genes encoding two enzymes in central carbon metabolism: phosphofructokinase (Pfk) in glycolysis and glucose-6-phosphate-dehydrogenase (Zwf) in the oxidative pentose-phosphate pathway. The former has two isoenzymes encoded by two genes (*pfkA* and *pfkB*). When growing cells on xylose, this rewiring ensures that cellular growth is dependent on carboxylation by Rubisco (Antonovsky et al., 2016) (Figure S2). Second, we heterologously expressed Rubisco, Prk, carbonic anhydrase (CA, which interconverts CO_2_ and bicarbonate), and FDH. Third, we grew our cells in xylose-limited chemostats, which maintain cells in constant starvation for organic sugar carbon. This growth medium allows cells to proliferate, which is essential for evolution to take place, but inhibits the flux through heterotrophic catabolic pathways. The chemostat also contained an excess of formate and was constantly sparged with CO_2_-enriched (10%) air. Thus, we created conditions where we predict that cells that accumulate mutations leading to diversion of flux to the autotrophic pathway are selected. Such cells will reduce their dependence on the external organic sugar carbon input and gain a large selective advantage compared to the non-mutated cells, which are limited by the supply of xylose. We used a dilution rate of 0.02 h^−1^ following our previous experience at evolving an *E. coli* synthesizing sugars from CO_2_ (Antonovsky et al., 2016) and our numerical and analytical analysis of minimal take-over times under competition and evolution in chemostats (Wides and Milo, 2018). We proceeded to test if, by continuous cultivation of the engineered strain in the tailored growth medium supplied by the chemostats, a completely autotrophic *E. coli* would evolve.

**Figure S3:**
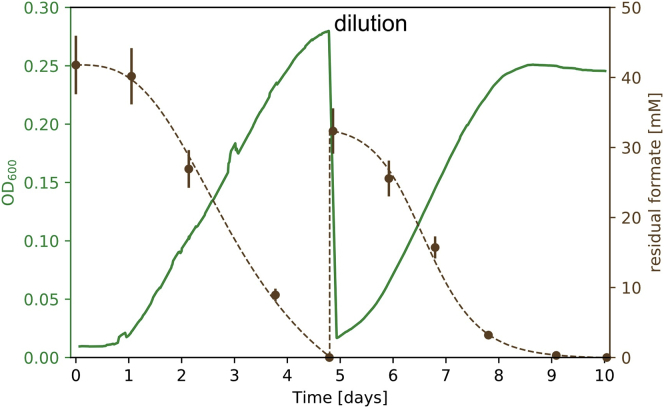
Growth Curve in Minimal Media with 35 mM Sodium Formate, Related to Figure 2 Repeated growth of the isolated evolved clone in liquid M9 minimal media with 35 mM sodium formate and sparging with a gas mixture of 10% CO_2_, 90% Air. The doubling time of the evolved cells at the given conditions is 18 ± 4 h. The residual concentrations of formate are shown in brown (n = 3, ± SD for values above 8 mM; n = 2, ± SD for values below 8 mM).

Upon the inoculation of the engineered strain into the xylose-limited chemostat with excess levels of formate, the residual levels of xylose dropped below the detection level, as expected under carbon-limited chemostat growth. We extracted samples from the chemostat once a week and tested for growth in autotrophic conditions. Specifically, these are chemo-organo-autotrophic conditions for *E. coli*, which consist of minimal M9 media supplemented with 30 mM sodium formate in an elevated CO_2_ (10%) atmosphere but without any other carbon source. Methylotrophs could potentially grow heterotrophically in such conditions, but we continuously monitored for the possibility of such contamination. After ≈200 days of chemostat propagation, equivalent to ≈150 chemostat generations, we observed growth in media devoid of xylose (autotrophic conditions). This phenotype persisted in all samples taken from that day on. Starting at day ≈350 of the chemostat adaptive laboratory evolution experiment, we omitted xylose from the feed media altogether as shown in Figure 2B. The sustained growth and turbidity implied full takeover by xylose-independent cells in the chemostat. We continued to validate growth of the extracted samples by repeatedly re-diluting them into fresh xylose-free media. The samples required elevated CO_2_ for growth, suggesting a carbon fixation growth mechanism. One of the isolated clones that showed more robust growth was chosen for in-depth characterization and exhibited a doubling time of 18 ± 4 h in autotrophic conditions, as shown in Figures 2C and S3. The cells had a formate-to-biomass conversion yield of 2.8 ± 0.8 gCDW/mol formate (Equation 1), similar to microorganisms that naturally grow autotrophically on formate (Pronk et al., 1991, Grunwald et al., 2015).

### Labeling by ^13^C Demonstrates that All Biomass Carbon Is Derived from CO_2_

**Figure 3:**
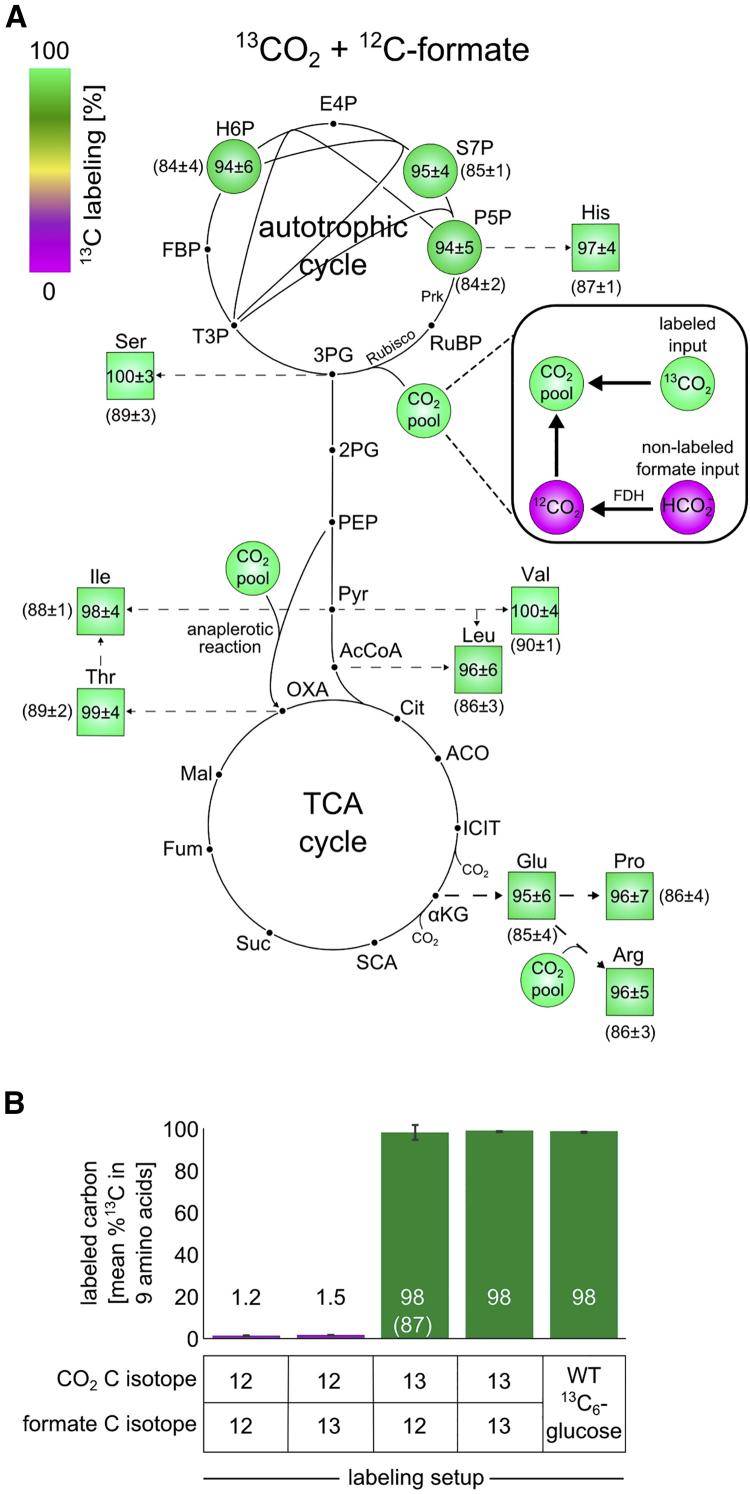
Isotopic Labeling Experiments Using ^13^C Show that All Biomass Components Are Generated from CO_2_ as the Sole Carbon Source (A) Values are based on LC-MS analysis of stable amino acids and sugar-phosphates (see STAR Methods). The fractional contribution of ^13^CO_2_ to various protein-bound amino acids and sugar-phosphates of evolved cells grown on ^13^CO_2_ and naturally labeled formate showed almost full ^13^C labeling of the biosynthesized amino acids. The numbers reported are the ^13^C fraction of each metabolite, taking into account the effective ^13^CO_2_ fraction out of the total inorganic carbon (which decreases due to unlabeled formate oxidation to CO_2_). The numbers in parentheses are the uncorrected measured values of the ^13^C fraction of the metabolites. Data are presented as mean ± SD; n = 5. (B) The average ^13^C fraction of nine analyzed amino acids of the evolved clone grown in different experimental setups. Experiments with ^13^CO_2_ as the substrate were carried in air-tight (i.e., “closed”) growth vessels. The bar with the parentheses represents the mean value after correction for the effective labeled fraction of CO_2_ in the experiment given the “pollution” with CO_2_ generated via formate oxidation and retention in the closed growth vessel. The value in the parentheses is the measured one, while the corrected value is shown without parentheses. As a positive control for maximal biomass ^13^C labeling, we grew wild-type *E. coli* in M9 minimal media supplemented with ^13^C_6_-glucose (far right). Error bars denote SD. See also Figure S4 and Tables S1, S2, and S3.

**Figure S4:**
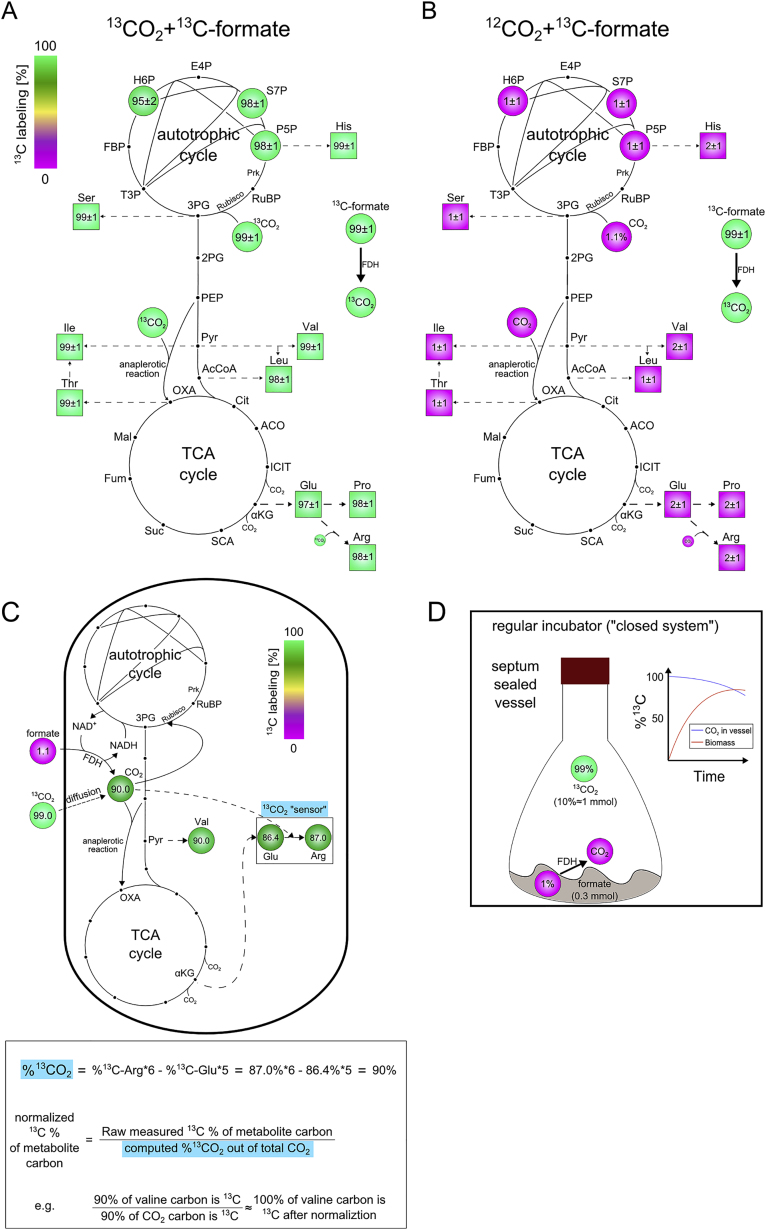
Amino Acid ^13^C Labeling Profile in Additional Labeling Experiments and the Effect of Unlabeled CO_2_ Emission from Formate Oxidation in Closed Vessels, Related to Figure 3 (A) The ^13^C fraction of various protein-bound amino acids and sugar-phosphates is close to 100% when the evolved cells were grown on ^13^CO_2_ and labeled ^13^C formate. The experiment was carried out in closed vessels (n = 3; ± SD). (B) The fractional contribution of ^13^C formate to various protein-bound amino acids and sugar-phosphates of evolved cells grown on ^12^CO_2_ and labeled ^13^C-formate showed minute ^13^C labeling of the sugar-phosphates and biosynthesized amino acids. The experiment was carried out in gas permeable vessels (n = 3; ± SD). (C) The weighted average of the effective isotopic composition of CO_2_ during a labeling experiment that starts with 99% ^13^CO_2_ (≈1 mmol) in the headspace and ≈0.3 mmol naturally labeled formate can be computed from the measured labeled fractions of glutamate and arginine, which we define as a ^13^CO_2_ “sensor.” The bottom box describes the calculation method and its implementation in the subsequent normalization of the raw labeling measurements of various metabolites (e.g., valine). (D) The experimental setup of isotopic biomass labeling with ^13^CO_2_ consists of a septum-sealed 250 mL growth flask and 10 mL of minimal M9 media with 30 mM naturally labeled sodium formate. In total, the vessel contains ≈0.3 mmol formate and ≈1 mmol of ^13^CO_2_ at the beginning of the experiment. The latter is flushed into the headspace via a thin needle, which is removed at the end of the flushing procedure. The initial inoculum of cells is also naturally labeled. As the cells grow and oxidize the formate to obtain energy, the isotopic composition of inorganic carbon within the vessel changes as depicted in the plot (blue line). The isotopic dynamics of the biomass carbon during autotrophic growth is depicted by the red line.

To test whether the evolved cells are indeed autotrophic and eliminate the possibility of unaccounted-for carbon sources or significant heterotrophic formate assimilation, we conducted comprehensive isotopic labeling experiments. First, we grew one of the evolved clones in an environment with ^13^C-labeled formate and ^13^CO_2_ for ≈10 generations (until isotopic steady state) and analyzed the ^13^C labeling patterns of various metabolites using LC/MS (Equation 3; Zamboni and Sauer, 2009). We observed that biomass building blocks across central metabolism had ≈98% of their carbon atoms labeled (Figures 3B and S4; Table S2). This is in line with the labeled formate and CO_2_ comprising ≈99% ^13^C and ≈1% unlabeled bicarbonate dissolved in the growth media. This provides definitive evidence that the cells’ biomass carbon is derived solely from CO_2_ and formate. To test whether formate is directly assimilated into biomass, the evolved cells were grown in minimal M9 media supplemented with ^13^C-labeled formate. The cultures were grown in a vessel with an air permeable cover inside a shaking incubator with elevated CO_2_ (10%, naturally labeled). The ^13^C labeling pattern of biomass building blocks following growth in this environment showed 1%–2% ^13^C labeling (Figures 3B and S4; Table S3), which is the value expected based on the natural abundance of ^13^C plus minor amounts of labeled formate being oxidized to ^13^CO_2_ and then fixed before equilibrating with the overall ^12^CO_2_ pool. These results demonstrate that the evolved cells essentially do not assimilate formate. One very minor exception is the incorporation of carbon from formate into one of the carbons of the purine rings. However, this is not a necessity of the *de novo* purine biosynthetic pathway but rather a technical issue, because the formyl moiety can either originate from formate, if it is present in the media, or from 10-formyl-tetrahydrofolate, which originates from serine (STAR Methods). The finding of negligible formate assimilation, together with the previous results indicating that there is no carbon source beyond CO_2_ and formate entering the biomass, serves as strong evidence that the evolved *E. coli* cells are indeed autotrophic.

In another validation experiment, we grew the cells in vessels with labeled ^13^CO_2_ and unlabeled formate. Because of the cost of ^13^CO_2_, this experiment is performed in closed vessels, which leads to some accumulation of unlabeled CO_2_ that is generated from oxidized formate, thus “polluting” the labeled ^13^CO_2_ pool. This can be monitored and corrected for by analysis of the labeling of glutamate (or proline) versus arginine, as the latter is produced from the former by the addition of CO_2_ (in the form of soluble bicarbonate; see Figure S4 and Equations 4 and 5 in STAR Methods). We observed that biomass building blocks across central metabolism had 85%–90% of their carbon atoms labeled. As shown in Figures 3A and 3B, when correcting for the effective labeling of intracellular CO_2_, the ^13^C-labeled fraction of the biomass building blocks is close to 100% (Table S1), showing in an independent and detailed manner the autotrophic nature of the evolved *E. coli*. All the labeling experiments described above were repeated both for cells from an isolated clone and on a mixed population sample from the chemostat, yielding practically identical results, depicted in Tables S1–S3.

### Laboratory Evolution Facilitated the Conversion to Autotrophy via a Relatively Small Number of Mutations

**Figure 4:**
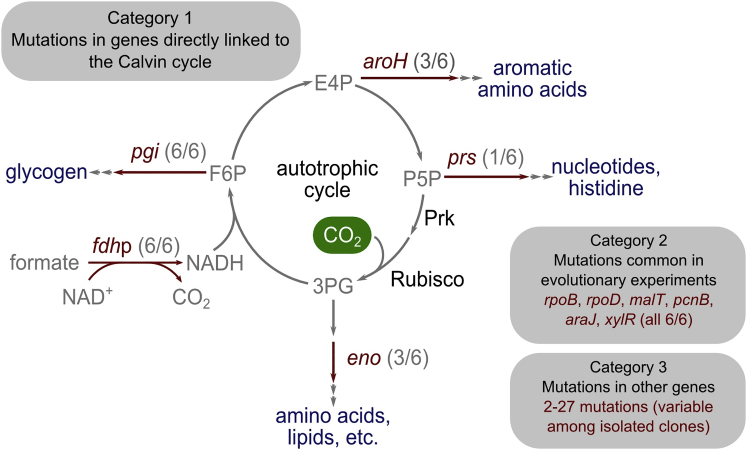
The Genetic Basis for Adaptation to Autotrophy The names of the mutated genes appear in red. The parentheses indicate the number of isolated clones in which the mutation appeared. As discussed in the main text, mutations observed across isolated clones can be divided into three broad groups. The first category includes mutations in genes with a direct metabolic link to the Calvin cycle, mostly flux branch points (the letter "p" at the end of the gene name denotes promoter region). The second category includes genes that are generic mutations common in other adaptive laboratory evolution experiments conducted with *E. coli*. The last category includes genes with uncharacterized role. Acronyms: E4P, erythrose-4-phosphate; P5P, pentose-5-phosphates; F6P, fructose-6-phosphate; 3PG, glycerate-3-phosphate. See also Table S4.

To better elucidate the genetic basis for the trophic-mode conversion to autotrophy, we isolated from the chemostat six clones capable of autotrophic growth on formate and sequenced their genome and plasmids (list of mutations specified in Table S4). Two of the clones were isolated while xylose was still present in the feed media (around day 250 of the evolutionary experiment, clones 1 and 2) and three after xylose was omitted from the chemostat feed media (around day 400 of the evolutionary experiment, clones 3, 4, and 5). A sixth clone was isolated after propagating one of the earlier isolated clones (clone 1) for several rounds of serial dilution (clone 6). Strikingly, as shown in Figure 4, we observed relatively few mutations fixed in the autotrophic clones (on top of the ancestral genetic background; see STAR Methods). We divided the mutated genes into three broad categories as described below.

The first category consists of genes encoding enzymes with a direct metabolic link to the function of the Calvin cycle. In line with previous analysis showing the need to balance the flux branch (bifurcation) points from autocatalytic cycles to ensure stable biomass production (Barenholz et al., 2017), we found a mutation in *prs* (I171T), the main flux branch point of the Calvin cycle in clone 6. This gene, which encodes ribose-phosphate-diphosphokinase, diverts ribose-phosphate toward biomass. Mutations in this enzyme were shown to play a crucial role in the kinetic stabilization of the Calvin cycle in *E. coli* by reducing the rate of D-ribose-5-phosphate efflux out of the cycle (Antonovsky et al., 2016, Herz et al., 2017). An additional key flux branch point is the gene *pgi*, encoding for glucosephosphate isomerase, whose inactivation was already shown to be important for stable operation of the synthetic Calvin cycle in a previous study from our group (Herz et al., 2017). In some of our isolated autotrophic clones (3–5), the *pgi* gene is completely absent, along with 16 other genes as part of a large (≈22 kb) chromosomal deletion. In the remaining clones, a single point mutation (H386Y, clones 1, 2, and 6) was identified in one of the catalytic residues at the active site (Totir et al., 2012), likely leading to down modulation of the enzyme activity. Overall, the modulation of Pgi activity, either by gene deletion or active site mutation, is common to all autotrophic clones isolated from the chemostat. Beyond these two previously reported flux branch points, we observed mutations in genes of two additional flux branch points. The first is *aroH* (mutation D109N in clones 1 and 6; and A68E in clone 2), which encodes a 2-dehydro-3-deoxyphosphoheptonate aldolase, the first committing step in the chorismate pathway leading to the biosynthesis of aromatic amino acids from erythrose-4-phosphate and phosphoenolpyruvate. All the clones without a mutated *aroH* gene contain a mutation in the enolase-encoding *eno* gene (G17V, clones 3–5), which could also be considered an extension of a bifurcation point affecting the flux diverted off the Calvin cycle. Overall, all our autotrophic isolated clones had mutations in more than one flux branch point, consistent with the mutations required for the stabilization of the Calvin cycle in *E. coli* in our earlier work (Antonovsky et al., 2016, Herz et al., 2017). Within the energy module, we observed in the promoter region of the plasmid-encoded *fdh* gene, either an 8-base pair or a 5-base pair deletion, which can affect the expression level of FDH to tune the rate of NADH production with the reducing power consumption rate by the Calvin cycle. The fact that we observed two independent mutations occurring in the same promoter region of *fdh* suggests a functional role for these mutations. However, FDH activity assays of crude extracts of WT *E. coli* BW25113 transformed with the mutated (8-base pair deletion variant) and non-mutated plasmids showed no significant difference in activity. Future work could help elucidate whether the mutations in the promoter region of *fdh* are indeed necessary for the phenotype and what is their physiological effect.

A second category of mutated genes consists of those commonly observed to be mutated in previous adaptive laboratory evolution experiments (Phaneuf et al., 2019). Members of this group include *pcnB* (R161P) (Masters et al., 1993), *rpoB* (D866E) (Utrilla et al., 2016), *rpoD* (F563S) (Malhotra et al., 1996), *malT* (E359K) (Gresham and Hong, 2015), and *araJ* (W156^∗^) (Reeder and Schleif, 1991). These mutations are suggested to be attributed to generic selective pressures found in long-term lab evolution experiments in minimal media and not necessarily to be specifically geared for the autotrophic phenotype. Similarly, the mutation in the *xylR* gene (Song and Park, 1997), encoding the regulatory protein for operons responsible for the catabolism of the sugar D-xylose (E337K), is probably related to the long period of xylose starvation in the chemostat but is irrelevant under the final autotrophic growth conditions. This is in line with observed mutations in the xylose catabolism operon in previous evolution experiments conducted in xylose-limited chemostats that were found to be not essential for the phenotype of the evolved strain (Antonovsky et al., 2016, Herz et al., 2017). Further supporting the notion that the above-mentioned mutations are generic mutations common to adaptive laboratory evolution experiments, all of them were fixed in the entire chemostat population during the first 130 days of the evolution, long before the appearance of the autotrophic phenotype. Therefore, they are most likely connected to the starvation state in which the cells were present during the evolution rather than directly related to the autotrophic phenotype. Nevertheless, some of these mutations might be linked to the emergence of the autotrophic phenotype through their global regulatory functions, such as in the case of the core transcription machinery (*rpoB*/*rpoD*). In addition, mutations in *pcnB*, which encodes poly(A) polymerase I, were reported to decrease the copy number of ColE1 plasmids (Masters et al., 1993, Pontrelli et al., 2018a). Because pFDH has a ColE1 origin of replication, the *pcnB* mutation most likely reduces the copy number of this plasmid to decrease the cellular burden associated with its maintenance and gene expression.

The last category of mutated genes includes mutations that currently have no characterized role and may be hitchhiker mutations. Across the different isolates, there are anywhere between 2 and 27 extra mutated genes, some of which could be refinement mutations (Quandt et al., 2014) of the autotrophic phenotype but are not strictly essential for it. Future research into the genetic underpinning of the autotrophic phenotype will help determine which of the observed mutations is essential and sufficient for synthetic autotrophy.

## Discussion

In this study, we demonstrate that it is possible to convert the trophic mode of an obligate heterotroph to full autotrophy over laboratory timescales through expression of heterologous genes combined with metabolic rewiring and laboratory evolution. This rapid trophic mode transformation showcases the outstanding plasticity of metabolism and demonstrates the power of the framework described here for designing and implementing the rewiring of cellular metabolism. The applied approach combines rational design with laboratory evolution that focuses on coupling cellular fitness to the desired functionality. The rationale behind this coupling is that predicting all possible constraints on the implementation of new metabolic functions is limited by insufficient information regarding the kinetics and regulation of the relevant components. Therefore, instead of attempting to rationally design components that comply with all the possible constraints, we created a rewired metabolic configuration and applied selective conditions under which the desired metabolic function is linked to fitness (Sauer, 2001). This link allows us to harness the power of natural selection for fine-tuning the metabolic network such that it would accommodate the new metabolic function (Blount et al., 2012). Because the cellular fitness of the rationally designed strains is linked to the activity of the introduced metabolic pathway, if this metabolic pathway is initially not active, the ancestor strain would not be able to grow, and laboratory evolution could not occur. To bridge this gap, an important component of the approach is the use of chemostats (Antonovsky et al., 2016). When using chemostats to grow cells continuously, the supply of limiting amounts of a surrogate substrate (e.g., xylose), which compensates for the lack of full activity of the introduced pathway, allows cells to slowly grow and facilitates the evolutionary process. The feedback that chemostats inherently implement keeps the surrogate substrate at very low concentrations, and thus maintains a continuously strong selective pressure to integrate the non-native pathway (Sonderegger and Sauer, 2003, Marlière et al., 2011, Wides and Milo, 2018).

The innovative potential of synthetic biology has led to an explosion of interest in leveraging recent advances toward sustainability challenges (French, 2019). One of the most important challenges is the assimilation of atmospheric CO_2_ for the production of food, fuels, and biochemicals (Kubis and Bar-Even, 2019). Although much progress has been made in recent years (Keller et al., 2013, Mattozzi et al., 2013, Antonovsky et al., 2016, Schada von Borzyskowski et al., 2018), all previous attempts at integrating synthetic CO_2_ assimilation pathways in non-native hosts have met limited success. Achieving synthetic autotrophy in a central biotechnological organism such as *E. coli* sets an important milestone toward the sustainable production of chemicals from CO_2_. Currently, growing our autotrophic *E. coli* strain on formate as an energy source leads to an overall net CO_2_ production (because formate is oxidized to CO_2_ at a higher rate than at which CO_2_ is assimilated into biomass [Figure S1]). Yet, in future coupling to a renewable energy source, formate would be produced electrochemically from CO_2_ (Innocent et al., 2009) with negative greenhouse gas emissions. Formate could then be used as the feedstock for biotechnological production of various chemicals using the synthetically autotrophic *E. coli* as the bioproduction platform. Integration of existing synthetic metabolic bio-production modules (Pontrelli et al., 2018b) into a synthetic autotrophic strain can expand its metabolic capacities in a modular fashion. In parallel, the autotrophic strain reported here can serve as a robust and genetically amenable model microbial organism for optimizing the components of the Calvin cycle such as the key carboxylating enzyme Rubisco (Aigner et al., 2017). This study is, therefore, a stepping stone to future efforts seeking to understand evolutionary transitions and harnessing synthetic biology on the path to more sustainable bioproduction.

## STAR★Methods

### Key Resources Table

**Table:** 

REAGENT or RESOURCE	SOURCE	IDENTIFIER
**Bacterial and Virus Strains**		

*E. coli* strain BW25113	Coli Genetic Stock Center (CGSC)	CGSC#: 7636
*E. coli* strain JW3887-1 Δ*pfkA* knockout	CGSC	CGSC#: 10802
*E. coli* strain JW5280-1 Δ*pfkB* knockout	CGSC	CGSC#: 11288
*E. coli* strain JW1841-1 Δ*zwf* knockout	CGSC	CGSC#: 9537

**Chemicals, Peptides, and Recombinant Proteins**		

Sodium formate BioUltra, ≥ 99.0% (NT)	Sigma-Aldrich	Cat. # 71359
Carbon dioxide (^13^C, 99%) (< 2% ^18^O)	Cambridge Isotope Laboratories	CLM-185-5
Sodium formate-^13^C (99 atom % ^13^C)	Sigma-Aldrich	Cat. # 279412
Streptomycin	Sigma-Aldrich	Cat. # S9137
Kanamycin	Sigma-Aldrich	Cat. # K1377
Chloramphenicol	Sigma-Aldrich	Cat. # C0378
D-xylose	Sigma-Aldrich	Cat. # W360600
D-glucose (U-^13^C_6_, 99%)	Cambridge Isotope Laboratories	CLM-1396-PK
Formate dehydrogenase from *Candida boidinii*	Sigma-Aldrich	Cat. # F8649
SPRIselect reagent	Beckman Coulter	Cat. # B23317
beta-nicotinamide adenine dinucleotide hydrate, 98+% (NAD+)	ACROS organics	Cat. # 124530010

**Critical Commercial Assays**		

Formic Acid Assay Kit	Megazyme	K-FORM
DNeasy blood & tissue kit	QIAGEN	Cat. # 69504
Nextera DNA Library Prep Kit	Illumina	Cat. # FC-121–1031
Pierce BCA Protein Assay Kit	Thermo Fisher Scientific	Cat. # 23225
BugBuster ® ready mix	Merck Millipore	Cat. # 71456
KAPA HiFi HotStart ReadyMix (2X)	KAPA Biosystems	KK2611

**Deposited Data**		

DNA sequencing raw reads	This paper	ENA study PRJEB34901
Code for constraints-based modeling and analysis of autotrophic *E. coli*	This paper	https://gitlab.com/elad.noor/sloppy/tree/master/rubisco

**Experimental Models: Organisms/Strains**		

*E. coli* strain BW25113	CGSC	CGSC#: 7636
Engineered *E. coli* strain BW25113 Δ*pfkA*Δ*pfkB*Δ*zwf* pCBB(Cm^R^, Amp^R^) pFDH(Strep^R^)	This paper	N/A

**Oligonucleotides**		

*pfkA* deletion validation forward primer	Sigma-Aldrich	N/A
5′-AGGGAGGGTAAACGGTCTATG-3′		
*pfkA* deletion validation reverse primer	Sigma-Aldrich	N/A
5′-CTTGCGGGTATATGTTGAGGG-3′		
*pfkB* deletion validation forward primer	Sigma-Aldrich	N/A
5′-TTAGCGTCCCTGGAAAGGTAAC-3′		
*pfkB* deletion validation reverse primer	Sigma-Aldrich	N/A
5′-TCCCTCATCATCCGTCATAGTG-3′		
*zwf* deletion validation forward primer	Sigma-Aldrich	N/A
5′-AGAGACTCACGGGTAATGAC-3′		
*zwf* deletion validation reverse primer	Sigma-Aldrich	N/A
5′-TTCTATCCGGGCGAGATAAG-3′		
Forward Primer for sequencing the promoter region of the *fdh* gene in the pFDH plasmid	Sigma-Aldrich	N/A
5′-TATTTACGCAGACCCAGTTC-3′		
Reverse primer for amplification of the promoter region of the *fdh* gene in the pFDH plasmid	Sigma-Aldrich	N/A
5′-TACAAATGTACGGCCAGC-3′		
Nextera index kit	Illumina	FC-121-1011

**Recombinant DNA**		

pCBB plasmid	Antonovsky et al., 2016	GenBank: KX077536
pFDH plasmid	This paper	Addgene plasmid #131706
pCP20 plasmid	Cherepanov and Wackernagel, 1995	N/A

**Software and Algorithms**		

Breseq	Barrick et al., 2014	https://barricklab.org/twiki/bin/view/Lab/ToolsBacterialGenomeResequencing
MassLynx v4.1	Waters	https://www.waters.com/waters/en_US/MassLynx-MS-Software/nav.htm?locale=en_US&cid=513662
Maven	Clasquin, Melamud and Rabinowitz, 2012	http://genomics-pubs.princeton.edu/mzroll/index.php
Python version 2.7	Python Software Foundation	https://www.python.org
COBRApy package for python v 0.17.1	Ebrahim et al., 2013	https://pypi.org/project/cobra/
Seaborn library for python v. 0.71	Michael Waskom	https://seaborn.pydata.org/

**Other**		

CO_2_ incubator with built in shaker	eppendorf	New Brunswick S41i
Fermenter for continuous culture	New Brunswick Sceintific	Bioflo 110
DASbox® Mini Bioreactor System	eppendorf	Cat. # 76DX04MB
DASGIP MX4/4 Gas Mixing Module for 4 Vessels with a Mass Flow Controller	eppendorf	Cat. # 76DGMX44
ACQUITY UPLC H-Class PLUS System	Waters	waters.com
Xevo TQ-S Mass spectrometer	Waters	waters.com
Thermo Q-Exactive mass spectrometer	ThermoFisher Scientific	https://www.thermofisher.com/il/en/home.html
Agilent 1200 series HPLC	Agilent Technologies	https://www.agilent.com/en/products/liquid-chromatography

### Lead Contact and Materials Availability

Further information and requests for resources and reagents should be directed to and will be fulfilled by the Lead Contact, Ron Milo (ron.milo@weizmann.ac.il). The pFDH plasmid generated during this study was deposited to Addgene (#131706). The pCBB plasmid as well as the autotrophic *E. coli* clones generated during this study are available from the Lead Contact upon completing a Materials Transfer Agreement.

### Experimental Model and Subject Details

#### *Escherichia coli*

We generated an engineered ancestor strain for chemostat evolution based on the *Escherichia coli* BW25113 strain (Grenier et al., 2014). We used P1 transduction (Thomason et al., 2007) to transfer knockout alleles from the KEIO strain collection (Baba et al., 2006) to our engineered strain, and to knock out the genes phosphofructokinase (*pfkA* and *pfkB*) and 6-phosphate-1-dehydrogenase (*zwf*). Following the transduction of each knockout allele, the Km^R^ selection marker was removed by using the FLP recombinase encoded by the pCP20 temperature-sensitive plasmid (Cherepanov and Wackernagel, 1995). Loss of the selection marker and the temperature-sensitive plasmid were validated by replica-plating the screened colonies and PCR analysis of the relevant loci. The engineered Δ*pfkA* Δ*pfkB* Δ*zwf* strain was then transformed with the pCBB plasmid (Antonovsky et al., 2016) (GenBank: KX077536) and with a pFDH plasmid (Addgene plasmid #131706) with a constitutive promoter controlling the expression of the *fdh* gene. Following whole-genome sequencing, we noted that the ancestral strain possessed the following three mutations - *fusA* T125I, *lrhA* Δ9 bp (85-93/939), and integration of a mobile insertion sequence (IS) element into the promoter region of the *xylE* gene (−21, position 4,232,204). These mutations were acquired during early handling of the strain prior to chemostat inoculation.

### Method Details

#### Generation of recombinant plasmids

To create the pFDH plasmid, an *E. coli* codon optimized DNA sequence based on the amino acid sequence of formate dehydrogenase from the methytholotrophic bacterium *Pseudomonas sp. 101* (Popov and Lamzin, 1994, UniProt: P33160) was synthesized and cloned with an N-terminal his-tag into a pZE21-MCS plasmid (Expressys, Germany). We replaced the P_LtetO-1_ promoter with a constitutive one driving medium transcription levels (clone #10 from Braatsch et al. [2008]) and a strong ribosome binding site (rbs B of Zelcbuch et al. [2013]). We replaced the Km^R^ selection marker on the plasmid with the *aadA* gene, which confers resistance to streptomycin. Details regarding the pCBB plasmid are reported in Antonovsky et al. (2016).

#### Preparation and utilization of growth media

Plasmid cloning and genomic modifications were carried out on a Luria Bertani medium with the relevant antibiotics (kanamycin (50 μg/ml), chloramphenicol (30 μg/ml, dissolved directly in the autoclaved M9 media and then filtered through a 0.22 μm PVDF filter) and/or streptomycin (100 μg/ml)). Engineered and evolved strains were grown on M9 minimal media supplemented with trace elements and the relevant carbon source(s). In the ^13^C-labeling experiments and for accurate estimation of growth parameters of the evolved cells on formate as the only organic compound, we used HPLC-grade water (Sigma Aldrich) and omitted EDTA from the trace elements.

The trace elements components and their concentrations in the M9 media are: 50 mg/L EDTA (omitted during ^13^C labeling experiments and growth measurements), 31 μM FeCl_3_, 6.2 μM ZnCl_2_, 0.76 μM CuCl_2_·2H_2_O, 0.42 μM CoCl_2_-6H_2_O, 1.62 μM H_3_BO_3_, 81 nM MnCl_2_·4H_2_O.

#### Growth tests of autotrophic *E. coli* strain

The growth experiments were conducted in a DASBox mini fermentation system (Eppendorf, Germany). The starting volume of each bioreactor was 150 mL M9 media supplemented with 30 mM or 35 mM sodium formate (Sigma Aldrich) as the carbon source, and trace elements (without the addition of EDTA and vitamin B1). Bacterial cells were seeded from a 15 mL starter at an OD_600_ of 0.12-0.14 (resulting in a 1:10 dilution by volume). Growth temperature was set to 37°C, and the chemostat was aerated at a rate of 6 L/hr with 90% air supplemented with 10% CO_2_. Values from the various probes were logged at 5 min intervals and used for analysis as described below. Once a day, 2 mL samples were removed from the bioreactor and used for media analysis (after filtration through a 0.22 micron PVDF Millex-GV syringe filter unit (Merck Millipore)) and for offline OD measurements (see below). Once the culture reached the stationary phase, ≈15 mL of the media were resuspended in fresh M9 media, as above, to a total of 150 ml, and the growth test was repeated.

Optical density measurements were performed online, using the integrated DASGIP® OD4 module and sensors. The values were converted into OD_600_ by taking samples from the growth medium at various optical densities and measuring the OD_600_ of each sample offline with a spectrophotometer (Ultrospec 10 Cell density meter, Amersham Biosciences) and a standard 10 mm polystyrene cuvette (Sarstedt, Germany).

We fitted a linear relation between the DASGIP® OD4 measurements and the OD_600_ measurements. After diluting the cells, the DASGIP® OD4 module was calibrated to give a value of 0 at the beginning of the second growth test. In this case, we fitted a linear relation between OD_600_ measurements of samples from the culture and the readings of the DASGIP® OD4 sensor, using the same slope as the one employed for the linear fit from the first growth test. Growth rates were determined by transforming OD_600_ measurements into logarithmic scale with a base of 2 and then calculating the growth rate over a sliding window interval of 150 sample points, in each window fitting a linear relation between log_2_(OD_600_) and time (in hours). The slope of each fit represents the estimated growth rate (in doublings per hour). We then calculated the average of the highest growth rate in the four experiments (two growth cycles for each of the two formate concentrations - 30 mM and 35 mM) to give our best estimate of the maximal growth rate. The doubling time was calculated as the inverse of the growth rate, expressed in units of hours per doubling. To estimate the uncertainty of our calculated growth rates due to the calibration error, we sub-sampled from the data to get 100 different linear relations (slopes and intercepts) between the DASGIP® OD4 measurements and the OD_600_ measurements. For each sampled set of parameters, we calculated the growth rate based on the same procedure described above. We used the mean and standard deviation of these 100 growth rates as our best estimators of the growth rate and its standard deviation in each growth test. We propagated the calibration error in each experiment assuming the calibration error is correlated across experiments.

#### Yield calculation for autotrophic growth

The yield was calculated based on the values of samples taken during the exponential phase of the growth according to the following equation:

(Equation 1) $$Y=\frac{B\left(t\right)-B\left(t_{0}\right)}{S\left(t\right)-S\left(t_{0}\right)}$$

where *B* is the biomass weight in units of gram cell dry weight (gCDW) and *S* is the amount of formate in units of moles. The biomass weight was inferred from the measured optical densities of the samples at 600 nm (OD_600_) via the conversion factor from OD_600_ to gCDW, which ranges between 0.3 gCDW × L^-1^ per OD_600_ for *E. coli* cells (Glazyrina et al., 2010) to 0.5 gCDW × L^-1^ per OD_600_ (Folsom and Carlson, 2015). We used the mean value of 0.4 ± 0.1 gCDW × L^-1^ per OD_600_ for the conversion.

#### Formate uptake rate calculation

Throughout each of the four growth experiments, we measured the concentration of formate in the growth medium at different time points by using both HPLC and an enzymatic assay (see Analysis of media composition section). We fitted the measured formate concentration over the course of each growth experiment with a four parameter logistic function of the form:

(Equation 2) $$y\left(t\right)=\frac{a-d}{1+{\left(\frac{t}{c}\right)}^{b}}+d$$

We then calculated the derivative of the fitted logistic function at each time point during the course of the growth to estimate the total formate consumption rate. The formate consumption rate was normalized to the amount of cellular biomass by using the OD_600_ of the culture at the same time point, and converting it to dry cellular mass assuming a factor of 0.4 gCDW × L^-1^ per OD_600_. We report the mean uptake rate and its standard error across the four different growth tests (two growth cycles for each of the two formate concentrations - 30 mM and 35 mM).

#### Chemostat evolution experiment

The evolution experiment was conducted in a Bioflo 110 chemostat (New Brunswick Scientific, USA) at a working volume of 0.7 L and a dilution rate of 0.02 h^-1^ (equivalent to a doubling time of ≈33 hours) at 37°C. The chemostat was fed media containing 4 g/L sodium formate and 0.5 g/L D-xylose as sole carbon sources. This amount of xylose in the feed makes xylose the limiting nutrient for cell growth in the chemostat. On days 47,166, 214, and 343 of the evolution experiment, the level of D-xylose in the feed media was reduced to 0.28, 0.13, 0.05, and 0 g/L, respectively. The concentration of formate was increased to 6 g/L on day 357, after the autotrophic growth phenotype was observed, and chloramphenicol (30 mg/L) and streptomycin (100 mg/L) were added to the feed media. Aeration of the chemostat was done through a DASGIP MX4/4 stand-alone gas-mixing module (Eppendorf, Germany) with a composition of 10% CO_2_ and 90% air at a flow rate of 40 sL/hr. To monitor the chemostat, a weekly sampling protocol was performed. Samples were taken for media analysis and phenotyping (inoculation of the bacteria on minimal media containing formate and lacking D-xylose). We calculated the biomass dependency metric of each sample as the ratio between the xylose carbon concentration (g carbon/L) in the feed and the carbon concentration in the culture biomass. The biomass carbon concentration was calculated with a conversion factor of 0.2 g carbon per 1 OD_600_ (Glazyrina et al., 2010, Folsom and Carlson, 2015). The optical density of each extracted sample was measured using a spectrophotometer (Ultrospec 10 Cell density meter, Amersham Biosciences) and a standard 10 mm polystyrene cuvette (Sarstedt, Germany).

For the following time points, only biomass concentration values are reported in the paper and not the metric of biomass dependency, which requires steady chemostat operation: Days 0-4, when the culture was grown in batch mode; days 134-137, when an especially low OD was measured for unknown reasons (possibly due to a malfunction) and the calculated biomass dependency values are thus extremely, and probably artificially, high (11 and 25 xylose carbon/biomass carbon); days 167-195. A technical malfunction on day 167 led most of the culture to be flushed out of the chemostat. The chemostat was switched to batch mode to enable recovery with fresh media. Until day 190, the OD remained low and thus a glycerol stock sample taken on day 166 was used as an extra inoculum. On day 195, the chemostat mode was restored.

#### ^13^C Isotopic labeling experiment

A culture of evolved cells grown on naturally labeled sodium formate in an elevated CO_2_ (10%, naturally labeled) incubator (New Brunswick S41i CO_2_ incubator shaker, Eppendorf, Germany) were diluted 8-fold into fresh M9 media with either 30 mM ^12^C or ^13^C-formate sodium salt (Sigma Aldrich) to a total volume of 10 mL of culture. In the “open” labeling setup, growth was carried out in 125 mL glass shake flasks with breathable sealing sticker-films (AeraSeal, Excel Scientific, USA), which allow free exchange of gases between the headspace of the growth vessel and the gas mixture of the incubator. The flasks were placed inside an elevated CO_2_ (10%) shaker-incubator (New Brunswick) with 37°C. After ≈3 doublings, the cells were again diluted 8-fold into fresh media of the same type. This procedure was repeated several times for at least 10 doublings within each of the conditions. Then, the cells were harvested for subsequent analysis of protein-bound amino acids and intracellular metabolites. In the “closed” labeling setup, growth was carried out in 250 mL glass shake flasks with a transparent extension, which allows the measurement of the optical density of the culture without opening it. After ≈3 doublings, the cells were diluted 8-fold into flasks covered with an air-tight rubber septa (SubaSeal, Sigma Aldrich). Then, the headspace of the flask was flushed with a gas mixture containing 10% ^13^CO_2_ (Cambridge Isotope Laboratories, USA) + 90% air or 10% ^12^CO_2_ + 90% air generated by a DASGIP MX4/4 stand-alone gas-mixing module (Eppendorf, Germany). The flasks were then placed in a 37°C shaker incubator. This procedure was repeated several times for at least 10 doublings for each of the conditions. Then, the cells were harvested for subsequent analysis of protein-bound amino acids and intracellular metabolites. The glass flasks used in the labeling experiments were pretreated by heating in a 460°C furnace for 5 hours to evaporate any excess carbon sources that could remain in the vessels from previous utilizations. Number of replicates (growth flasks) in each condition with the evolved isolated clone: (a) ^13^CO_2_ + ^13^C-formate (n = 3). (b) ^13^CO_2_ + ^12^C-formate (n = 5). (c) ^12^CO_2_ + ^13^C-formate (n = 3). (d) ^12^CO_2_ + ^12^C-formate (n = 1 for this trivial control). Number of replicates (growth flasks) in each condition with a sample taken from the chemostat after day 350: (a) ^13^CO_2_ + ^13^C-formate (n = 3). (b) ^13^CO_2_ + ^12^C-formate (n = 2). (c) ^12^CO_2_ + ^13^C-formate (n = 3). The labeling of WT *E. coli* BW25113 cells using U^13^C_6_-glucose was performed with n = 1 of this well established control.

#### Sample preparation for LCMS analysis

After harvesting the biomass, culture samples were prepared and analyzed as described in Antonovsky et al. (2016). Briefly, for protein-bound amino acids, ≈3 mL of culture at OD_600_ turbidity of ≈0.1-0.15 were pelleted by centrifugation for 5 minutes at 8,000 g. The pellet was suspended in 1 mL of 6N HCl and incubated for 24 hours at 110°C. The acid was subsequently evaporated with a nitrogen stream, resulting in a dry hydrolysate. Dry hydrolysates were resuspended in 0.6 mL of MilliQ water, centrifuged for 5 minutes at 14,000 g. The supernatant was then injected into the LCMS. Hydrolyzed amino acids were separated using ultra performance liquid chromatography (UPLC, Acquity - Waters, USA) on a C-8 column (Zorbax Eclipse XBD - Agilent, USA) at a flow rate of 0.6 mL/min and eluted off the column using a hydrophobicity gradient. Buffers used: A) H_2_O + 0.1% formic acid and B) acetonitrile + 0.1% formic acid with the following gradient: 100% of A (0-3 min), 100% A to 100% B (3-9 min), 100% B (9-13 min), 100% B to 100% A (13-14 min), 100% A (14-20 min). The UPLC was coupled online to a triple quadrupole mass spectrometer (TQS - Waters, USA). Data was acquired using MassLynx v4.1 (Waters, USA). Amino acids and metabolites used for analysis were selected according to the following criteria: We chose amino acids that have peaks at a distinct retention time and m/z values for all isotopologues and also showed correct ^13^C labeling fractions in control samples that contained protein hydrolyzates of WT cells grown with known ratios of ^13^C_6_-glucose to ^12^C-glucose.

For intracellular metabolites, ≈8 mL of culture at OD_600_ turbidity of ≈0.1-0.15 were pelleted by centrifugation for 5 minutes at 5,000 g. The pellet was suspended in 4 mL of a cold (−20°C) acetonitrile:methanol:water (40:40:20) extraction solution and incubated overnight at this temperature. The next day, the extracts were centrifuged (5 minutes at 16,000 g), and the supernatant was transferred into fresh tubes. Organic solvents were subsequently evaporated using a speedvac vacuum concentrator. The aqueous phase was evaporated by freeze drying. Dry extracts were stored at −80°C until the mass spectrometry analysis. Prior to injection into the mass spectrometer, the dry extracts were suspended in 200 μL of a 1:1 methanol:water solution, centrifuged (5 minutes at 16,000 g) and then the supernatant was transferred to a vial for injection. Metabolites were separated using liquid chromatography. A ZIC-pHILIC column (4.6 mm × 150 mm, guard column 4.6 mm × 10 mm; Merck) was used for liquid chromatography separation via a gradient elution with a solution of 20 mM ammonium carbonate, with 0.1% ammonium hydroxide, and acetonitrile at 0.1 mL/min. Detection of metabolites was performed using a Thermo Scientific Exactive high-resolution mass spectrometer with electrospray ionization, examining metabolites in a polarity switching mode over the mass range of 75–1,000 m/z. The identities of the compounds were verified by matching masses and retention times to a library of authenticated standards. Data analysis was performed using the Maven software suite (Clasquin et al., 2012).

#### Isotopic analysis composition of biomolecules

The total ^13^C fraction of each metabolite was determined as the weighted average of the fractions of all the isotopologues of the metabolite, as depicted in the equation below:

(Equation 3) $$f_{13_{C}}=\frac{\sum_{i=0}^{n}f_{i}\times i}{n}$$

where *n* is the number of carbons in the compound (e.g., for the amino acid serine, n = 3) and f_*i*_ is the relative fraction of the *i*-th isotopologue that contains *i* ^13^C carbon atoms.

#### Calculation of the effective ^13^C fraction

We used the carbamoyl-phosphate moiety as a marker for the isotopic distribution of the intracellular inorganic carbon pool. Carbamoyl-phosphate is generated by carbamoyl phosphate synthetase from bicarbonate as the carbon substrate. Carbamoyl-phosphate is then condensed with ornithine in the L-arginine biosynthesis pathway. We looked at mass isotopologue distribution of L-arginine, which contains an extra carbon from carbamoyl-phosphate (the guanidinium group carbon), versus the mass isotopologue distribution of either L-proline or L-glutamate, which are similar to that of ornithine. We calculated the effective ^13^C labeling of intracellular inorganic carbon (f_*13CO2, effective*_) by using the following equation (written for glutamate but can be equivalently used with proline instead):

(Equation 4) $$f_{13_{CO_{2'}}effective}=\sum_{i=0}^{6}f_{arg_{i}}-\sum_{i=0}^{5}f_{glu_{i}}$$

where f_*13CO2, effective*_ is the relative fraction of ^13^CO_2_ out of the total pool of CO_2_ (or more formally the inorganic C pool), and f_*arg_i*_ and f_*glu_i*_ are the fraction of the *i*-th isotopologue of arginine and glutamate respectively. We sum over all isotopologues (equal to the number of carbon atoms in the compound, 6 for arginine and 5 for proline or glutamate). We repeated the calculation using the measured isotopologue fractions of proline instead of those of glutamate. We used the average of those two calculations as a more robust estimator of the effective level of ^13^CO_2_ and the associated uncertainty. We then used the computed labeled fraction of ^13^CO_2_ to normalize the ^13^C-labeled fractions of all the measured metabolites using the following equation:

(equation 5) $$f_{13_{C_{met_{j},corrected}}}=\frac{f_{13_{C_{met_{j}},measured}}}{f_{13_{CO_{2}},effective}}$$

where *met*_*j*_ stands for the *j*-th measured metabolite (or protein-bound amino acid).

An analogous correction procedure using the labeled fractions aspartate and carbamoyl-aspartate was performed in a recent study (Bennett et al., 2008) to account for incomplete labeling owing to incorporation of non-labeled inorganic carbon in the media.

#### Whole-genome sequencing

DNA extraction (DNeasy blood & tissue kit, QIAGEN) and library preparation procedures were carried as previously described in Herz et al. (2017). Tagging and fragmenting (‘tagmentation’) using the Nextera kit (Illumina kits FC-121–1031) was performed by mixing 1 μL containing 1.5 ng of genomic DNA, 1.25 μL of TD buffer, and 0.25 μL of TDE1. The mixture was mixed gently by pipetting and placed for incubation in a thermocycler for 8 min at 55°C. Next, “tagmented” gDNA underwent PCR-mediated adaptor addition and library amplification by mixing 11 μL of PCR master mix (KAPA KK2611/KK2612), 4.5 μL of 5 μM index1 (Nextera index kit FC-121-1011), 4.5 μL of 5 μM index2, and 2.5 μL of tagmented DNA in each well. The final total volume per well was 22.5 μl. The thermocycler was run with the following program: 1) 72°C for 3 min, 2) 98°C for 5 min, 3) 98°C for 10 s, 4) 63°C for 30 s, and 5) 72°C for 30 s. 6) Repeat steps (3)–(5) 13 times for a total of 13 cycles. 7) 72°C for 5 min. 8) Hold at 4°C. PCR cleanup and size selection were done in several steps: mixing 12 μL of magnetic beads SPRIselect reagent (Beckman Coulter B23317) with 15 μL of each PCR reaction. Incubation at room temperature for 5 min followed by 1 min on a magnetic stand. The clear solution was discarded, and the beads were mixed with 200 μL of freshly made 80% ethanol. An ethanol wash was performed twice, and the plate was then incubated at room temperature for 5 min to allow for the evaporation of residual ethanol. The sample was eluted with 30 μL of ultrapure water for 5 min at room temperature, and the beads were removed using the magnetic stand.The prepared libraries were sequenced by a Miseq machine (Illumina). Analysis of the sequencing data was performed as previously described in Antonovsky et al. (2016) and Herz et al. (2017) using the breseq software (Barrick et al., 2014) with genomic and plasmid DNA sequences as references for alignments of sequencing reads.

To exclude the possibility of contamination in the different experiments, we extracted the DNA from bacterial pellets taken at the end of the experiments, sequenced them as described and validated that following alignment of the sequencing reads to the reference genome and plasmid sequences; at least 95% of the reads were aligned.

#### Analysis of media composition

Media samples collected during the evolution experiment and batch-growth experiments were first filtered through a 0.22 micron PVDF Millex-GV syringe filter unit (Merck Millipore), and stored at −80°C. After thawing, the media samples they were analyzed with an Agilent 1200 high-performance liquid chromatography system (Agilent technologies, USA) equipped with a refractive index detector and an anion exchange Bio-Rad HPX-87H column (Bio-Rad, USA). The column was eluted with 5 mM sulfuric acid at a flow rate of 0.6 mL/min at 45°C. Samples with a formate concentration below the detection limit of the HPLC were analyzed by an enzymatic assay kit (Megazyme, Ireland). Media samples from the evolution experiments were each measured once. Media samples from the batch growth experiments were measured 3 times, with the mean ± SD is shown in Figure 2C. The samples analyzed with the enzymatic kit were measured twice; the mean ± SD is reported.

#### Measurement of FDH activity in cell extracts

The assay was adapted from Berríos-Rivera et al. (2002) with several modifications listed below. In brief, both the original pFDH and the mutated one (Δ8 bp position 918) were transformed into TSS competent *E. coli* BW25113 cells by heat shock and selected on LB agar plates with streptomycin. Five colonies of each type were picked, verified by PCR and grown overnight at 37°C in 2 mL of M9 minimal medium supplemented with 50 μg/mL Streptomycin, 0.4% glucose and 30mM formate. When cells reached an OD_600_ of 1, they were harvested by centrifugation (15 minutes; 4000 g; 25°C) and pellets were lysed with 0.5mL BugBuster ® ready mix (Merck Millipore) for 25 minutes at room temperature. Crude extracts were then centrifuged for 30 min at 4000 g, 4°C to remove the insoluble fraction. Total protein concentrations were measured using a Pierce BCA Protein Assay Kit (Thermo Fisher Scientific) according to the manufacturer’s multi-well plate protocol. The enzymatic activity assay was performed in the presence of 200mM PBS PH-7.0, 10mM B-mercaptoethanol, 100mM sodium formate and 2mM NAD^+^ (ACROS organics). 190 μL of assay mix were added to 96-well plate, three wells (repeats) for each sample. The assay mix was pre-incubated at 37°C inside the plate reader for 15 min. All protein lysates were diluted to 0.1 mg/mL. 10 μL of lysate from each sample were injected into three replicate wells. The increase in NADH concentration resulting from formate oxidation was monitored at 340 nm (Infinite 200 Pro (Tecan, Switzerland)) over time. We averaged the slopes between replicates and compared the slope averages between bacterial lysates that originally contained the mutated plasmid to those with unmutated plasmids and observed no significant difference between them. The experiment included lysate from wild-type *E.coli* as a negative control and pure formate dehydrogenase (*C. boidinii*, Sigma-Aldrich) protein with the same overall concentration as a positive control.

#### *In silico* analysis of autotrophic *E. coli*

For our flux balance analysis of the *E. coli* strains, we used the Core *Escherichia coli* Metabolic Model (Orth et al., 2010a), and added the *rubisco*, *prk*, and *fdh* reactions (Can be accessed directly on GitLab). Then, the following changes were made to the model:

• PFK, ZWF (G6PDH2r in the code model), and PFL were knocked out
• The rate bounds for RBC, PRK, and FDH were set to the default values, i.e., to 0 - 1000 mmol/g/h
• All carbon-containing export/import reactions were removed, except for formate and CO_2_ (which were left unbounded, i.e., −1000 to 1000 mmol/g/h)
• We assumed that all formate uptake is done by diffusion, i.e., via the reaction FORt. Therefore, we set the bounds on FORt2 (formate proton symporter) to 0, and the bounds for FORt to 19 ± 2 mmol/g/h (based on the measured total formate uptake rates).
• Based on the measured values, the growth rate bounds were set to: 0.04 ± 0.01 h^-1^.

We then used the resulting model to generate a Phenotypic Phase Plane (Edwards et al., 2001). Such plots depict the feasible space where flux solutions exist given the flux balance analysis constraints. In Figure S1A, formate uptake rate is the controlled parameter (relaxing the constraint mentioned above), and the range of possible net CO_2_ production rates is shown on the y axis. The rate of FDH is completely determined by the formate uptake since it is the only reaction that can metabolise formate in the core model. The net CO_2_ production rate can still vary slightly depending on the growth rate (which is a function of how much of the CO_2_ is fixed by Rubisco).

Even when setting the formate uptake rate to the measured value (19 ± 2 mmol/g/h), there is still some redundancy in our flux solution space (due to the uncertainty ranges and also the stoichiometry itself). Therefore, we used the objective of minimum sum of fluxes (also known as parsimonious flux balance analysis, or pFBA) to get a unique flux solution (Holzhütter, 2004). For the minimum sum of fluxes solution, the growth rate is at its upper limit (0.05 1/h) and the formate uptake rate is at its lower limit (17 mmol/gCDW/h). The net production is calculated by the difference between all decarboxylating reactions and all carboxylating ones. We visualize this calculation by a stacked bar plot in Figure S1B. Since all energy and reducing potential comes from formate, we can see that FDH is responsible for almost all of the decarboxylations, and greatly surpasses the amount of carboxylations (mainly performed by rubisco) which sum up to 2.4 mmol/gCDW/h. Therefore, there is a positive net CO_2_ production of about 15 mmol/gCDW/h.

Finally, we were interested to see what would the net rate of carbon fixation be for our evolved strain when the formate is produced electrochemically from CO_2_. In this case, all CO_2_ produced by FDH cancels out, and the net CO_2_ *fixation* rate is 2.1 mmol/gCDW/h, where only about 13% of the carbon fixed by RBC and PPC is released as CO_2_ in PDH, ICD, and ME1 (Figure S1C).

All calculations were done using COBRApy (Ebrahim et al., 2013), and can be found in the following Jupyter notebook on our GitLab repository: https://gitlab.com/elad.noor/sloppy/tree/master/rubisco.

### Quantification and Statistical Analysis

Statistical details of individual experiments, including number of replicates is reported either in the figure legends or relevant method details. Data are presented as means with error bars indicating SD unless otherwise stated. The calculations were performed either with Microsoft Excel or with python.

### Data and Code Availability

The data that support the findings of this study are available from the corresponding author upon reasonable request. The Illumina short reads generated in this study have been deposited at the European Nucleotide Archive (ENA) with study accession number PRJEB34901. The python code for the *in silico* metabolic analysis of autotrophic growth is available on GitLab: https://gitlab.com/elad.noor/sloppy/tree/master/rubisco.

## Supplemental Information

Document S1. Tables S1–S3

Table S4. Mutations Identified in Evolved Clones, Related to Figure 4
