# Supplementary material for: Conversion of Escherichia coli to Generate All Biomass Carbon from CO2
Source: Cell. 2019 Nov 27;179(6):1255–1263.e12. doi: 10.1016/j.cell.2019.11.009 (PMC6904909; doi:10.1016/j.cell.2019.11.009)
Supplement: Document S1. Tables S1–S3 [file mmc1.pdf]

Cell, Volume *179*

Supplemental Information

Conversion of *Escherichia coli*

to Generate All Biomass Carbon from CO_2

Shmuel Gleizer, Roee Ben-Nissan, Yinon M. Bar-On, Niv Antonovsky, Elad Noor, Yehudit Zohar, Ghil Jona, Eyal Krieger, Melina Shamshoum, Arren Bar-Even, and Ron Milo

Table S1. ^{13}C-labeled fractions of protein-bound amino acids and sugar-phosphates following isotopic labeling experiments with $^{13}CO_2$ + ^{12}C-formate, related to Figure 3. The presented values are mean (±S.D.). *Values that after normalization slightly exceeded 100% were written as 100%.

Metabolite	Isolated Clone			Mixed Population		
	measured	normalized	repeats	measured	normalized	repeats
Ser	89.2 ± 2.6%	99.9 ± 3.1%	n=5	92.1 ± 2.7%	100* ± 3.5%	n=2
His	86.5 ± 1.3%	97.0 ± 3.9%	n=5	89.0 ± 3.3%	98.0 ± 3.4%	n=2
Val	89.6 ± 1.4%	100* ± 3.8%	n=5	92.4 ± 3.3%	100* ± 3.5%	n=2
Thr	88.5 ± 1.5%	99.3 ± 4.2%	n=5	90.2 ± 2.8%	99.3 ± 3.5%	n=2
Pro	85.9 ± 4.1%	96.3 ± 6.6%	n=5	87.2 ± 2.4%	96.0 ± 3.3%	n=2
Leu	86.0 ± 3.5%	96.4 ± 5.9%	n=5	87.5 ± 2.3%	96.3 ± 3.4%	n=2
Ile	87.9 ± 1.9%	98.5 ± 4.0%	n=5	89.8 ± 2.8%	98.8 ± 3.4%	n=2
Arg	86.0 ± 3.0%	96.4 ± 5.4%	n=5	87.1 ± 2.5%	95.9 ± 3.3%	n=2
Glu	84.7 ± 3.6%	95.0 ± 6.4%	n=5	86.0 ± 2.2%	94.6 ± 3.3%	n=2
S7P	84.8 ± 1.1%	95.1 ± 4.3%	n=5	82.7 ± 4.1%	91.1 ± 3.1%	n=2
P5P	83.9 ± 1.5%	94.1 ± 4.7%	n=5	80.7 ± 5.7%	88.9 ± 3.1%	n=2
H6P	83.5 ± 3.8%	93.7 ± 6.1%	n=5	81.8 ± 2.2%	90.0 ± 3.1%	n=2
AMP/ADP/ATP	77.2 ± 1.4%	86.6 ± 4.3%	n=5	74.0 ± 1.8%	84.1 ± 3.2%	n=2
UMP/UDP/UTP	87.1 ± 1.2%	97.6 ± 4.5%	n=5	84.2 ± 4.8%	95.7 ± 6.1%	n=2

Table S2. ^{13}C-labeled fractions of protein-bound amino acids and sugar-phosphates following isotopic labeling experiment with $^{13}CO_2$ + ^{13}C-formate, related to Figure 3. The presented values are mean (±S.D.).

Metabolite	Isolated Clone		Mixed Population	
	measured %^{13}C	repeats	measured %^{13}C	repeats
Ser	99.3 ± 0.1%	n=3	96.8 ± 1.4%	-
His	98.9 ± 0.2%	n=3	95.8 ± 1.6%	n=3
Val	99.2 ± 0.03%	n=3	97.5 ± 0.9%	n=3
Thr	98.5 ± 0.7%	n=3	93.6 ± 3.1%	n=3
Pro	97.7 ± 0.3%	n=3	92.4 ± 2.7%	n=3
Leu	98.2 ± 0.3%	n=3	93.2 ± 2.5%	n=3
Ile	99.1 ± 0.03%	n=3	97.5 ± 0.9%	n=3
Arg	97.6 ± 0.3%	n=3	92.3 ± 2.3%	n=3
Glu	97.4 ± 0.4%	n=3	91.0 ± 2.6%	n=3
S7P	97.9 ± 1.1%	n=3	98.4 ± 0.2%	n=3
P5P	97.9 ± 0.5%	n=3	97.3 ± 0.7%	n=3
H6P	95.0 ± 2.1%	n=3	96.0 ± 1.6%	n=3
AMP/ADP/ ATP	98.9 ± 0.3%	n=3	99.1 ± 0.2%	n=2
UMP/UDP/ UTP	99.7 ± 0.3%	n=3	99.2 ± 0.4%	n=2

Table S3. ^{13}C-labeled fractions of protein-bound amino acids and sugar-phosphates following isotopic labeling experiment with $^{12}CO_2$ + ^{13}C-formate, related to Figure 3
The presented values are mean (±S.D.).

Metabolite	Isolated Clone		Mixed Population	
	measured %^{13}C	repeats	measured %^{13}C	repeats
Ser	1.0 ± 0.5%	n=3	n/a	-
His	1.5 ± 0.1%	n=3	1.3 ± 0.1%	n=3
Val	1.5 ± 0.1%	n=3	1.3 ± 0.1%	n=3
Thr	1.4 ± 0.4%	n=3	1.6 ± 0.1%	n=3
Pro	1.7 ± 0.1%	n=3	1.4 ± 0.03%	n=3
Leu	1.4 ± 0.2%	n=3	1.1 ± 0.03%	n=3
Ile	1.3 ± 0.1%	n=3	1.2 ± 0%	n=3
Arg	1.8 ± 0.1%	n=3	1.9 ± 0.03%	n=3
Glu	2.0 ± 0.1%	n=3	2.1 ± 0.1%	n=3
S7P	1.2 ± 0.1%	n=3	1.6 ± 0.1%	n=3
P5P	1.2 ± 0.03%	n=3	1.2 ± 0.2%	n=3
H6P	1.3 ± 0.04%	n=3	1.4 ± 0.1%	n=3
AMP/ADP/ ATP	11.2 ± 0.3%	n=3	n/a	
UMP/UDP/ UTP	0.4 ± 0.3%	n=3	n/a	
